# Rapid Health Impact Assessment of a Proposed Poultry Processing Plant in Millsboro, Delaware

**DOI:** 10.3390/ijerph16183429

**Published:** 2019-09-16

**Authors:** Leah Baskin-Graves, Haley Mullen, Aaron Aber, Jair Sinisterra, Kamran Ayub, Roxana Amaya-Fuentes, Sacoby Wilson

**Affiliations:** 1 Maryland Institute for Applied Environmental Health, University of Maryland, 255 Valley Drive, College Park, MD 20742, USA; leah.baskin@gmail.com; 2 Environmental Science and Policy, University of Maryland, 0220 Symons Hall, College Park, MD 20742, USA; haley.mullen14@gmail.com (H.M.); aaronjaber@gmail.com (A.A.); 3 Public Health Science Program, University of Maryland, 255 Campus Drive, College Park, MD 20740, USA; jairsinisterra@gmail.com (J.S.); kamranayub4@gmail.com (K.A.); 4 Environmental and Occupational Health, George Washington University, 950 New Hampshire Ave, NW, 7th floor, Washington, DC 20052, USA; roxanaamaya@gwmail.gwu.edu

**Keywords:** health impact assessment, poultry processing plant, environmental justice

## Abstract

In 2013, Allen Harim Foods purchased the former site of a Vlasic Pickle plant in Millsboro, Delaware, and proposed to convert the site into a poultry processing plant that would process approximately two million birds weekly. This generated concerns about the proposed plant’s potential to impact health and quality of life among residents. We conducted a rapid health impact assessment (HIA) of the proposed plant to assess baseline environmental health issues in the host community and projected impacts. The scoping and baseline assessment revealed social, economic, and health disparities in the region. We also determined that residents in the area were already underserved and overburdened with pollution from multiple environmental hazards near the proposed plant including two sites contaminated with hazardous wastes, a power plant, and another poultry processing plant. The projected size and amount of poultry to be processed at the plant would likely cause increased levels of air, soil and water pollution, additional odor issues, and increased traffic and related pollution and safety issues. The information generated from the HIA formed the basis of a campaign to raise awareness about potential problems associated with the new facility and to foster more engagement of impacted residents in local decision-making about the proposed plant. In the end, the HIA helped concerned residents oppose the new poultry processing plant. This case study provides an example of how HIAs can be used as a tool to educate residents, raise awareness about environmental justice issues, and enhance meaningful engagement in local environmental decision-making processes.

## 1. Introduction

### 1.1. Poultry Production and Impacts on the Environment and Human Health

Industrial poultry production is widely known as a major environmental hazard [1,2,3,4,5]. Research has confirmed that concentrated poultry production results in significant air and water pollution [1]. Factory-style farming of chicken tends to create greenhouse gas emissions, eutrophication of nearby waterways, and large volumes of waste [4]. In terms of airborne pollutants, poultry production releases significant emissions of ammonia (NH_3_), methane, and sulfur dioxide [6]. Other pollutants such as volatile organic compounds (VOCs), particulate matter (PM) including PM_2.5_, and airborne nitrogenous compounds (e.g., nitrogen dioxide) can have deleterious health effects (both chronic and acute) including respiratory conditions (i.e., bronchitis, asthma in children), heart disease, and lung cancer [7,8,9,10,11,12].

Aside from air pollution concerns, industrial poultry farming can impact water quality as well [13]_._ Current practices used to manage livestock wastes do an insufficient job of protecting rivers, streams, and other water bodies from contaminants present in chicken waste [5,14]. Excessive amounts of nutrients can cause eutrophication and deaths of large populations of fish, due to hypoxia/anoxia and high levels of ammonia [14,15,16]. Moreover, the large volumes of waste produced by chicken farming are associated with elevated health risks for nearby residents, because poultry by-products typically contain pathogens, such as the Avian Influenza virus, *Salmonella,* and *Campylobacter* from contaminated blood, flesh, and excreta [5].

Another concern is antimicrobial resistant bacteria found in poultry production. The consumption of poultry products may lead to exposure to antimicrobial resistant pathogens increasing health risks. For example, Fluoroquinolone resistant *Campylobacter*—a foodborne zoonotic pathogen found in chickens—may cause enteritis and diarrhea [17]. Methicillin resistant *Staphylococcus aureus* (MRSA) continues to be a major concern in poultry production. MRSA has been detected in poultry products and having contact with chickens is a risk factor for MRSA colonization [18].

Exposure to waterborne contamination associated with chicken waste can occur in recreational settings (i.e., swimming or fishing from contaminated rivers), or during the ingestion of polluted groundwater or surface water used for human consumption [14,19,20,21,22,23,24,25]. The leaching of nitrates and pathogens into water can cause significant cognitive loss and nervous system impairment when ingested by humans [2]. Furthermore, eutrophication can spur the growth of toxic microorganisms, such as *Pfiesteria piscicida*, that have been found to cause temporary memory loss, immunosuppression, and decreased cognitive function in exposed populations [2,26,27,28], respiratory problems and eye irritation, as well as gastroenteritis, headaches, and fatigue [2,29,30]. Skin irritation and lesions have also been reported among those with direct contact with contaminated surface waters, particularly among fishermen [2,31].

### 1.2. Community Concerns about a Proposed Poultry Processing Plant in Delaware

In 2013, Allen Harim Foods bought a closed Vlasic Pickle facility (hereafter referred to as Harim Millsboro) in Millsboro, Delaware. Allen Harim planned to convert the former pickle plant into a chicken processing plant that would process two million chickens per week on an annual basis. Furthermore, the town of Millsboro is in Sussex County, a county ranked first in the United States for broiler chicken production. Annually, almost 600 industrial chicken farms in Sussex produce 200 million chickens for consumption [32]. Before they are sent to market, the 200 million chickens are trucked to one of seven specialized facilities in Delaware for processing. The new processing plant raised concerns from nearby residents over impacts on local air and water quality and human health. Residents also felt that the Harim Millsboro plant would exacerbate existing health issues associated with local pollution sources, such as two Superfund sites (i.e., sites contaminated with hazardous substances designated for cleanup by the US Environmental Protection Agency) and two processing plants.

For Harim Millsboro to begin operation, the Sussex County Planning and Zoning Commission and Board of Adjustments (BOA) had to review and approve the company’s request for an exception to zone the land as a potentially hazardous use. The change was approved by the BOA even though residents submitted 270 comments opposing the exemption request. As a result, residents felt that the County’s efforts of engaging the community in the zoning process violated many regulations. For example, a 2013 letter from the Socially Responsible Agriculture Project (SRAP) and Protecting Our Indian River (POIR), a local non-profit, to the County’s zoning office noted a number of violations including: (1) failure to provide written notice of the initial hearing to all parties of interest, and (2) a failure to notify or seek input from the Delaware Department of Health on the potential adverse health effects the facility may have on nearby residents including vulnerable populations.

Despite community concerns, the state of Delaware does not require the use of health impact assessments in the permitting process for poultry processing plants. The production site is, however, subject to several federal-level regulations that govern the waste produced by the facility. The Resource Conservation and Recovery Act (RCRA) and the Clean Water Act (CWA) regulate hazardous waste and water, respectively. In order to maintain operations, the poultry processing plant must comply with these policies by appropriately managing their effluent and meeting safe drinking water standards [33]. The Delaware Department of Natural Resources and Environmental Control (DNREC) is the agency responsible for monitoring compliance with these policies, which operates at the state level. This leads to a key issue with this process, which is that local and state health departments typically do not have any jurisdiction over the siting of poultry processing facilities. Instead, this is governed by environmental and natural resource agencies, such as DNREC, whose primary mission is not to protect public health.

### 1.3. Proposed Use of a Health Impact Assessment (HIA)

In consultation with SRAP, the Assateague Coastkeeper, and Food and Water Watch, the community contacted the Maryland Institute for Applied Environmental Health, School of Public Health, University of Maryland (UMD) to perform a health impact assessment (HIA) of the proposed facility. The National Academy of Sciences (NAS) defines an HIA as:

A systematic process that uses an array of data sources and analytic methods and considers input from stakeholders to determine the potential effects of a proposed policy, plan, program, or project on the health of a population and the distribution of those effects within the population. HIA provides recommendations on monitoring and managing those effects.

An HIA can also evaluate the potential benefits of a proposed policy, plan, program, or project [34]. An HIA also recommends actions that can be taken to manage the negative impacts. A rapid HIA is used to obtain input from a community about their concerns about the proposed project within a shorter time frame (approximately three months). A rapid HIA is also necessary when fewer resources including funding and staff support are available.

In consultation with SRAP, Food and Water Watch, and POIR, it was decided that instead of a more comprehensive HIA, a rapid HIA would be completed. There was not enough funding to perform a comprehensive HIA and time was an issue for stakeholders who believed decisions being made in the permitting process did not adequately respond to their concerns. In this paper, we will discuss the development of the rapid HIA, results of the HIA, and impacts of the HIA in decision-making related to building the chicken processing plant.

## 2. Materials and Methods

To assess the potential negative health impacts of the planned Harim Millsboro chicken processing plant, we followed the guidelines for a rapid HIA. A typical HIA has six steps including: (1) screening, (2) scoping, (3) assessment, (4) recommendations, (5) reporting, and (6) evaluation and monitoring. Due to limited resources and time, we only implemented the screening, scoping, assessment, recommendations, and reporting steps of the HIA. Conducting a rapid HIA allowed the team to determine possible exposures related to the plant, as compared with the standards deemed acceptable by the US Environmental Protection Agency (EPA). In addition, with this evaluation, we were able to examine other possible adverse effects and the possible economic benefits that the plant could have on the local community and surrounding area.

### 2.1. Screening

The screening process involves determining if an HIA is feasible, timely, and if it would add value to the decision-making process. Based on the time-sensitive nature of this situation, the team concluded that a rapid HIA would be appropriate, as it would allow stakeholders from the local community, advocacy organizations, and government agencies to receive information about the potential positive and negative impacts of the chicken processing plant quickly. Due to the presence of pre-existing environmental hazards, it was determined that the Harim Millsboro plant could contribute further to the cumulative burden that the community already faced. In addition, a vulnerable population that could experience especially high exposure to pollution and thus, increased adverse health effects, would be children, given the site’s proximity to local schools.

### 2.2. Scoping

The scope of the assessment was established based on input from residents who identified concerns and expert interviews on pollution in Millsboro. Researchers used US census data to examine the community’s sociodemographic characteristics, Delaware Health and Social Services information on community health, and US EPA data on emissions from local facilities and on exposure to air and water pollutants present in the community. Researchers identified pollutants of concern, levels present in media based on US EPA data or reports, as well as health impacts associated with those pollutants. Geographic Information Systems (GIS) were used to map environmental hazards in the area of concern. QGIS software was also used to spatially examine the distribution of hazards and their proximity to different sociodemographic groups at the census block group level (i.e., percentage non-White, percentage in poverty, percentage of homeownership, percentage less than high school education). 

### 2.3. Assessment

In this phase of the HIA, the team performed a baseline assessment of environmental hazards and pollution issues; demographic analysis of populations who may be impacted by the operation; assessment of health status for local populations; and evaluated possible negative and positive impacts of the operation on the environment and human health—particularly impacts on vulnerable populations. Information for the baseline assessment was obtained through secondary analysis of government reports, non-governmental reports, and peer-reviewed literature. 

To establish a baseline profile of residents near the proposed site, the team collected information on race, income, employment status, educational attainment, and access to healthcare services. In addition, baseline community statistics regarding cancer, pulmonary issues, all-cause and disease-specific mortality rates, and birth outcomes (e.g., birth weight) were also collected to identify health issues in the community of concern. To properly determine the individual and cumulative exposure that would be associated with opening the new facility, a summary of existing environmental hazards in the community was established. This information included contaminants associated with each site and the potential health effects of exposure to each toxicant, including those that are connected specifically to large poultry processing plants. To supplement the findings and determine the total impact of the Harim Millsboro plant, including its construction, factors such as increased traffic, foul odors, and possible occupational health concerns were examined. The US EPA EJSCREEN tool, an environmental justice mapping tool, was used to examine air pollution risks associated with traffic. Meanwhile, peer-reviewed literature and government reports were reviewed to assess impacts related to foul odors and possible occupational health issues.

### 2.4. Reporting

Results of the HIA were presented to Protecting Our Indian River (POIR), along with a survey to assess the concerns and awareness of other residents. A summary factsheet was also produced.

## 3. Results

### 3.1. Geographic Analysis of Millsboro, Delaware

Millsboro is located along the Indian River in southeastern Delaware, about 25 miles northwest of Ocean City, Maryland and nearly 21 miles southeast of Rehoboth Beach, Delaware. Three communities of concern (Colonial Estates, Holiday Acres, and Possum Point), are adjacent to the proposed processing plant site and lie within the zip codes of interest (namely 19,966 and 19,939). These communities located in the southeastern portion of Millsboro, border Whartons Branch stream and the Indian River. Near the proposed site (<2-mile radius), there are many residences including 240 townhouses, 247 trailer homes, 533 single-family houses, and 45–60 apartments. There are also two schools near the proposed processing plant.

### 3.2. Sociodemographic Analysis

The percentage of families living below the Federal Poverty Level (FPL) in the census block group for our three neighborhoods of concern (Colonial Estates, Holiday Acres, and Possum Point) was 19.89%, significantly higher than the family poverty rates for Sussex County (8.9%), Delaware (7.8%), and the USA (11.3%). The median household income in the same area was only $37,434 [35]. This was lower than the income level of Sussex County ($52,710), Delaware ($59,878), and the USA ($53,046). The area not only has limited economic capital, but also limited health care infrastructure. Sussex County has been designated as a health professional shortage area (HPSA) and medically underserved area (MUA) by the Health Resources and Services Administration [36].

According to a spatial assessment of the distribution and concentration of industrial chicken farms, poultry plants were in areas (i.e., census tracts) with moderate numbers of non-White residents (16%). There was also a disparity in the homeownership rate. The 56.8% homeownership rate was lower than the county, state, and national rates. Education was also a factor taken into consideration; the percentage of residents with less than a high school education (15.9%) was higher than levels for the county, state, and country.

### 3.3. Baseline Environmental Assessment

**Figure 1:**
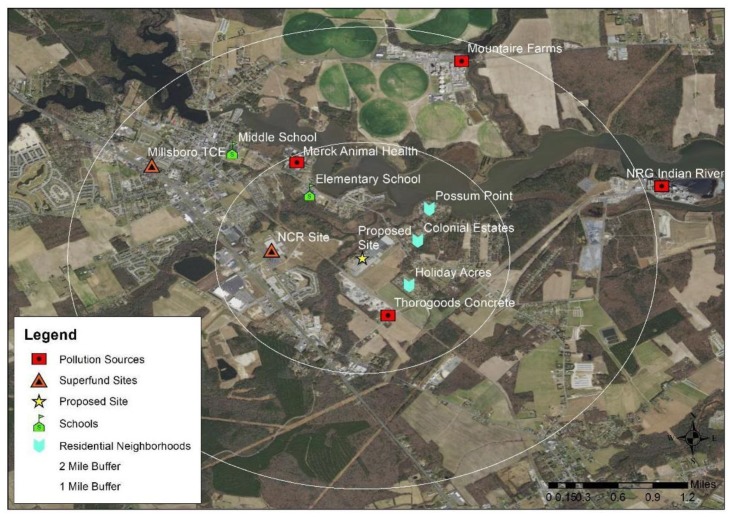
Map of Environmental Hazards Near the Millsboro Community.

We used Google Earth to visually examine the presence of environmental hazards near the proposed site of the Harim chicken processing plant (<2 miles) (Figure 1). We found there were four industrial facilities including a power plant and another chicken processing plant, two Superfund sites, two schools, and three residential areas (Possum Point, Colonial Estates, and Holiday Acres). A summary of some of these existing hazards is presented in Table 1.

### 3.4. Chemicals of Concern

Table 2 provides an overview of chemicals of concern identified by residents during the screening and scoping phases of the rapid HIA, along with chemicals found at high levels at the site of the proposed poultry processing plant. 

### 3.5. Baseline Health Assessment

#### 3.5.1. Respiratory Issues and Healthcare Access

An analysis of 2013 health status data for the Millsboro zip codes of interest shows that the percentage of residents with cancer, chronic bronchitis, emphysema, and heart disease (all types) was noticeably higher than rates experienced at the County, state, and national levels [50]. Health statistics from Geographic Research, Inc indicate that 13.0% of residents had heart diseases (all types) compared to 11.9% statewide [50]. In 2013, the rate of emphysema was 2.44% for the Indian River region, higher than rates for Delaware (2.16%) and the USA (2.12%) [50]_._ These statistics indicate that introducing another facility within the zip codes could possibly increase disease and mortality rates due to higher PM levels and emissions of other toxic chemicals. 

Clean Air Task Force data from 2014 on adverse health effects estimated that 7500 deaths occur nationwide per year due to PM emissions from power plants [51]. This represented a reduction from 13,000 deaths nationwide per year in 2010, and researchers attributed it to policies such as the Mercury Air Toxics Rule and the Cross State Air Pollution Rule, which contributed to nationwide decreases in sulfur dioxide emissions (68%) and nitrogen oxide emissions (55%). Data on the Indian River region shows that exposure to PM was associated with deaths, cardiovascular disease, and respiratory issues in the community. PM exposure accounted for 375 combined cases of illness and more than $139 million in costs. Estimates for Sussex County were lower at 141 combined cases and $9.4 million in costs.

We obtained useful data on access to healthcare resources in Millsboro from the United States Health Resources and Services Administration (HRSA). HRSA designated Sussex County as a medically underserved area (MUA), with a shortage of mental health professionals (more than 30,000 residents per provider), dentists (ratio of more than 5000 residents to one dentist), and primary health care providers (ratio of more than 3500 residents per one provider), who provide care to low-income residents [36].

#### 3.5.2. Cancer

In 2007, the Delaware Department of Public Health (DPH) examined cancer incidence rates from 2000–2004 in the Indian River area (six zip codes adjacent to the NRG Indian River power plant). The study showed that the age-adjusted cancer rate for the Indian River region was 553.9 cases per 100,000, which was significantly higher than the cancer rate for Delaware (501.3), and the entire US (473.6) [52]. Lung cancer represented a statistically significant percentage of cases. The study also found that the lung cancer incidence rate was much higher for Indian River (105.6) compared to Sussex County (79.1), Delaware (76.9), and the USA (63). However, this study lacked data on socioeconomic status, exposure levels, access to health care, smoking habits, and length of time having resident in the area.

In response, DPH administered the Indian River Community-Level Survey (IRCLS) to collect data on tobacco use and other risk factors from residents [53] in 2009_._ In the self-report survey, 71 participants had been diagnosed with lung cancer, with 44% from the Indian River area. The study revealed that current smokers were 17.5 times more likely to develop cancer than non-smokers, and residents who had smoked were 10.5 times more likely to develop lung cancer than residents who had never smoked. The study also found statistical significance among smoking status and occupation; participants who worked in a high-risk industry (e.g., construction, chemical, agricultural, manufacturing, or pharmaceutical) were 3.4 times more likely to develop lung cancer compared to participants who did not work in those industries, regardless of smoking [54]. The study concluded that Indian River participants were significantly more likely than non-Indian River participants to be heavy smokers and to have worked in a high-risk industry, regardless of cancer status. The IRCLS also had a small sample size, and factors such as genetic predisposition could have negatively affected the study results [53].

### 3.6. Impact Assessment

#### 3.6.1. Exposures Related to Poultry Processing, Transport, and Waste Discharge

The health of residents can be significantly impacted by the entire process of chicken production including: (1) raising chickens in confinement buildings, (2) transporting chickens to processing plants for slaughter, and (3) shipping the processed meat out of the plant to customers. During transport, bird crates can fall onto roadways if not properly secured. Unfortunately, an entire crate of chickens can be lost if the truck has an accident and is overturned [54]. The arrival of poultry transport trucks to the processing plant can spread harmful bacteria into the environment potentially exposing other drivers, workers, and residents to these harmful bacteria [54]. For example, one study detected increased levels of antibiotic-resistant bacteria on surfaces and in the air inside of passenger vehicles that drove behind these trucks [55]_._

Research has shown that approximately 0.25%–0.86% of chickens die prior to arriving for processing due to poor handling, stress, and extreme temperatures [56]. These preprocessed carcasses are disposed of through composting, incineration, or landfill. As a result of these disposal methods, pollutants can be added to the soil, air, and water. Water in large quantities is used at processing plants, creating an issue with poultry processing wastewater (PPW) treatment and disposal, and conserving local water supplies. For the Harim facility, which expects to process two million birds weekly and produce 200 tons of offal/day, about two million gallons of water would be used daily. Large quantities of water are also used to transport offal out of processing areas, where PPW is then discharged to waterways or land applied. PPW can contain high concentrations of chlorine, nitrogen, and phosphorus [57]. In addition, the use of antibiotics in poultry feed can cause PPW to contain antibiotic-resistant bacteria [58]. As PPW is discharged, runoff into local rivers and streams and infiltration into groundwater can cause antibiotic-resistant bacteria to contaminate sources of drinking water. This means that individuals who live close to these facilities and consume water from drinking water wells may be exposed to PPW-related contaminants.

#### 3.6.2. Odor Issues

During scoping, many residents strongly expressed concerns about odors from the proposed chicken processing plant. Grease, fats, blood, and carcasses in the PPW act as a medium for bacterial growth during processing. This can result in decay [59]. Decaying PPW contains organic particulate matter, volatile fatty acids, sulfurous compounds such as mercaptans and H_2_S, and nitrogenous compounds such as NH_3_, that are emitted into the air as malodorous compounds. H_2_S has a smell like rotten eggs, and mercaptan gas is often added to butane or propane gas for cooking and the odor smells like rotten cabbage. These noxious odors act as a chronic nuisance for nearby residents.

Millsboro residents already experience odors from one processing plant (Mountaire Millsboro). In 2013, Mountaire released almost 3200 pounds of toxic chemicals to the ambient air (76% H_2_S). Concerned stakeholders believed that adding a second plant within two miles of an existing plant would greatly increase the presence and intensity of noxious odors. For example, one resident stated that the stench was unbearable on certain days, preventing him from going outdoors. Research has shown that residents living near concentrated animal feeding operations (CAFOs) reported lower measures of quality of life, more anger and fatigue, and decreased mood because they felt unable to go outside or open the windows in their homes due to the lack of clean, odor-free, fresh air [60,61,62,63].

#### 3.6.3. Occupational Health

The health of processing plant workers is important in the poultry processing industry, especially since many of these workers are of lower socioeconomic status. As a result, they might lack information on the potential hazards they face [64]. The Bureau of Labor Statistics notes that these workers face demanding conditions, monotonous tasks, and potential injury from using sharp tools and slipping on wet floors [65]. The Occupational Safety and Health Administration (OSHA) identified other risks, including electrical hazards and exposure to avian flu for workers [66].

The risk of injury and infection is particularly acute in poultry slaughterhouses. A study of self-reported poultry slaughterhouse injuries recorded by OSHA found high rates of skin lacerations on the hands and skin infections in workers who handle sharp tools or handle the birds themselves [67]. Other studies found a decline of lung function of workers due to exposure to organic dusts, endotoxins, and ammonia [68,69]. Finally, musculoskeletal problems have been identified in poultry processing workers due to long periods spent on their feet and in awkward positions [64].

#### 3.6.4. Traffic Concerns

The Harim Millsboro facility proposed to process two million chickens weekly. This would involve the transport of chickens via truck to and from the plant 47 times per day [70]. Harim Millsboro planned to hire 700 employees which would have added 300–700 vehicles per day traveling to and from the plant. Most areas of Millsboro are between the 60th and 80th percentiles in the country for proximity to traffic (results from US EPA EJSCREEN), posing a significant health threat to the many residents who live near the areas in which these vehicles will be traveling. This additional traffic may elevate safety risks for two schools near the facility. During the summer, the area of concern is congested with visitors and residents traveling on Route 113, a main thoroughfare that runs through Millsboro, to nearby beaches for vacation. Additional trucks and vehicles will likely increase traffic congestion, pose additional abrasion to the roads, degrade air quality, and present safety concerns to residents living along the Iron Branch Road corridor and within school zones.

Increased diesel truck traffic may also lead to elevated exposure to diesel exhaust. Diesel exhaust contains over 40 toxic air contaminants including cancer causing agents (e.g., arsenic, formaldehyde, benzene) [71]. It is possible that the levels of PM, CO, CO2, and nitrogen oxides in the air will increase significantly due to an increase in vehicle emissions and dust from poultry litter. Furthermore, due to more vehicles on the road transporting chickens from the farms to the processing plant, there may be an increase in runoff pollution. This runoff pollution will contain dirt, dust, engine oil, antifreeze, fertilizers, and pesticides. Rainwater and melting snow will carry these contaminants and wash off into nearby streams, rivers, and soil [72]. This runoff could impact water quality and increase health risks for nearby residents who rely on private drinking water wells.

#### 3.6.5. Economic Benefits of the Proposed Processing Plant

Delaware’s poultry industry provides a few economic benefits including the employment of as many as 3500 individuals and supports as many as 2900 supplier positions that rely on the poultry industry [73]. Moreover, Allen Harim Foods, planned to bring 165 new jobs into the Millsboro region in order to support the new chicken processing plant [74]. Previously, in 2010, the Delaware Economic Development Office announced that Mountaire Farms invested $34.5 million in its poultry complex in Millsboro [75]. This corporation and its operations were reported to employ over 3600 people in Sussex County [75]. Growth in the chicken processing sector enables more overall economic growth because it directly affects the demand of other industries within Delaware. The poultry industry supports Delaware’s grain industry, as Delmarva’s chicken industry purchased $980 million in grain crops to support the increasing demand for chicken feed [73]. In 2016, the poultry industry drove $3.3 billion dollars in total economic revenue, as a result of hundreds of family farms and large processing companies, such as Mountaire and Perdue farms [73]. It is possible that the new facility would add to the total economic revenue in the County and state. The economic benefits of the facility could be viewed as a positive offset to potential negative environmental and social externalities related to the construction and operation of the facility. 

## 4. Discussion

### 4.1. Key Findings

Even without the opening of a large-scale poultry processing plant, Millsboro residents are overburdened with pollution from multiple sources near the proposed processing plant. The existing pollution stems from a coal-fired power plant, an animal vaccine factory, a concrete factory, two US EPA Superfund sites, and an existing poultry processing plant. The site of the processing plant is a brownfield site with contamination due to the presence of H2S, arsenic, nitrates, VOCs, trichloroethylene (TCE), chromium, cobalt, chloride, and particulate matter.

In addition to poor health outcomes, degradation of air and water quality may occur due to releases associated with the projected size of the plant and the amount of chickens that would be processed annually. Vehicles used by employees who work at the plant or used to transport chickens to and from the facility may increase traffic-related air pollution (TRAP) and traffic congestion problems. Additionally, large amounts of water used at the facility may result in animal wastes in the water, potentially contaminating local rivers and streams and posing a threat to groundwater supplies. Furthermore, ambient odor problems burdening residents due to the presence of a processing plant currently in operation could be worsened by the construction and operation of the Harim facility.

Since the rapid HIA is the first of its kind in assessing the impacts of a chicken processing plant in the United States, we did not have other HIAs on this topic for comparison. However, we believe that this processing facility will have similar impacts on the environment and human health that have been seen with industrial chicken production and transport. Industrial chicken farms are sources of organic dust, microorganisms in manure, litter, dust, air, and emissions from volatile odorous compounds (VOCs). Poultry workers are exposed to higher levels of organic dust compared to individuals working in swine or cow farms [76]. Organic dust affects workers’ respiratory systems and may cause chronic respiratory symptoms, decreased lung function, rhinitis, and eczema among poultry workers with more than five years or occupational exposure [76]. Methods of transporting poultry to slaughterhouses are another route of exposure for motorists and neighborhoods on the transportation pathway [55]. Animals are transported in pens, open cages, or crates stacked on trailers with little to no containment which does not provide a barrier between pathogens and the environment and the cages are highly contaminated with bacteria and feces [77]. Antibiotic resistant bacteria, especially *Staphylococcus aureus*, are a common issue and have become a cause of global morbidity and mortality [78]. *Staphylococcus aureus* found at processing plants can contaminate poultry products placing consumers at risk for foodborne illnesses and necrotizing skin infections, folliculitis, and toxic shock syndrome [79].

### 4.2. Challenges and Limitations Related to the Implementation of the Rapid HIA

There were numerous challenges with performing the rapid HIA. One challenge was that the HIA was completed as part of a capstone project. The main researcher who performed the HIA was only able to commit one semester to complete the HIA and had limited prior experience with or knowledge of the HIA framework. This meant that the student researcher had to spend some of the time getting on the job training on the HIA framework through guidance from their advisor. Additionally, since this was a capstone project, the student researcher did not have ample support from other researchers to help execute the HIA. This was particularly an issue for the scoping phase of the project. More support in the scoping phase of the project could have increased opportunities to engage stakeholders including concerned residents, health officials, local government representatives, and industry representatives who were not interviewed for the HIA.

Another challenge was the distance between the researcher who primarily executed the HIA and the area of concern. The researcher during the development of the HIA was a student at the University of Maryland-College Park, which is located 2 h away from Millsboro, the site of the planned chicken processing plant. Logistically, the time and distance between the student researcher and the area of concern made attending community meetings related to the siting of the facility difficult and scheduling in-person meetings for the scoping and assessment phases of the HIA difficult as well. One related challenge was the lack of resources to perform a deeper assessment of potential impacts and benefits of the chicken processing plant. There was an initial plan to perform a health survey of local residents who would be impacted by the operation, but the lack of financial resources to cover costs of such a survey and time needed to execute the survey in advance of the permitting decision made this idea unfeasible. The logistical issues meant the HIA would not include any new data collected for the assessment phase but only leveraged secondary data in the form of peer-reviewed literature, government reports, and independent non-peer reviewed research studies. In addition, many residents whose main source of drinking water was from the wells located near the processing plant had concerns about the quality of their water, but the time and resources needed to perform adequate water quality testing were not available.

Additionally, the fact that this was a voluntary HIA requested by concerned stakeholders but not required by law was an additional limitation. The state of Delaware does not require the use of a health impact assessment in permitting processes for poultry processing plants. Harim Foods did commission an outside consultant, BP Environmental, Inc. to prepare an Environmental Site Assessment report, a limited subsurface report, and a brownfield investigation report in 2013, as part of a response to the state-level National Environmental Protection Act (NEPA) process. If the HIA was required as part of a separate assessment process or included as part of a required NEPA assessment (i.e., environmental impact statement (EIS)), there may have been opportunities to secure more resources to execute the HIA including staff time and funding to cover a health survey, focus groups, air pollution monitoring, and risk assessments that could have made the HIA more robust and comprehensive. Additionally, being a legally binding document could mean that any alternatives or recommendations provided to mitigate, reduce, or eliminate impacts of the new facility on human health would need to be explored and implemented in accordance with the law.

### 4.3. Impact of the HIA

One of the most important aspects of the HIA is the dissemination of results to stakeholders of concern. During the reporting stage, information obtained through the HIA can be shared with impacted residents who then use this information to leverage additional assistance and support from advocacy groups, government agencies, and public officials. In July 2015, HIA results were presented to POIR members and other meeting attendees along with a one-page fact sheet listing some of the primary processing plant pollutants for quick and easy reference at the Indian River Senior Center.

Although the meeting was generally well-attended by concerned residents, those with decision-making power were not present. There were no local or state-level elected officials, and only one person from an agency came. Although the state health department was specifically invited to attend, they were not present, either. James Brunswick, member of the Committee Involvement Advisory Committee for the Delaware Department of Natural Resources and Environmental Control (DNREC) attended the meeting, though the regulatory agency did not offer any community support. The meeting mostly served as verification for the community members’ concerns about the processing plant, and many locals expressed their misgivings about the disproportionate levels of pollution that the processing plant would add to their community.

In October 2015, Allen Harim Foods decided to terminate their plans to build a new poultry processing plant in Millsboro at Vlasic site. The HIA supported community concerns that the plant could contaminate the Millsboro area and worsen environmental health in a region with several environmental hazards. The information was used by the local community in partnership with the Assateague Coastkeeper and Food and Water Watch to launch an educational campaign about the proposed facility and help organize local citizens to be engaged in local decision-making. The plant would have potentially exposed residents to several contaminants including particulate matter, arsenic and VOCs and other issues including water pollution and traffic-related hazards.

The HIA also made a substantial impact on Delmarva Poultry Industry (DPI), the poultry trade association to which Allen Harim belongs. In 2016, DPI’s executive director sent a letter to administrative officials in the state of Maryland including the Maryland University System Chancellor, University of Maryland (UMD) President, the Board of Regents, and Deans for the School of Public Health and College of Agriculture and Natural Resources. In addition, the letter was sent to the General Assembly Eastern Shore delegation, the Governor, and the Secretary of Agriculture. The letter reflected DPI’s dismay at the University of Maryland’s involvement in Delaware’s business interests. Furthermore, the letter claimed that the HIA was an “attack” on the Delmarva chicken industry, and that the University appeared to be working against the poultry enterprise. The executive director asked that the University of Maryland issue an official disclaimer for the HIA if it was not being endorsed by the University.

The use of political pressure and intimidation by industry representatives is not unusual in industrial animal agriculture. In North Carolina, researchers studying the impacts of industrial hog farming on nearby residents received pressure to break confidentiality agreements with participants, due to a public records request from the North Carolina Pork Council [80]. The research team received lukewarm support from the University of North Carolina as it tried to protect the rights of local communities. In addition, there has been intimation of residents who live near these operations with physical violence and threats of job loss for workers employed at industrial hog operations and processing plants [80,81]. Regardless of the political pressure and intimidation from the chicken industry in Delaware, the HIA had positive impacts on residents. 

### 4.4. Value of the HIA in the US and beyond 

This study emphasizes the value of the HIA process in environmental decision-making in the United States. Entities such as Pew Charitable Trust, the Centers for Disease Control and Prevention (CDC), the United States EPA, and the National Academy of Sciences (NAS) have developed reports, guidelines, and toolkits on the implementation and evaluation of HIAs. Many of these HIAs have focused on the built environment, air pollution, improving access to resources for physical activity, addressing social determinants of health, environmental injustice, and health disparities [82]. For example, the CDC identified the impacts of 23 HIAs, and the results illustrated that the decision-makers would have made significantly different decisions without the input from the HIAs [82]. The National Conference of State Legislators reported that 55 bills in 17 states contained elements in support of HIAs between 2009 and 2014 [83]. In fact, in 2007, Alaska chose to institutionalize the use of HIAs in their natural resources permitting process [84]. Massachusetts also mandated HIAs in transportation-related projects by passing the Healthy Transportation Compact [84]. The use of HIAs are becoming increasingly more popular since they were introduced in the US 15 years ago, as they empower community members to address social determinants of health and help provide protection against health disparities.

The HIA framework has also been used extensively internationally [85]. Through efforts of the World Health Organization (WHO) and other governmental agencies in Asia, Africa, Europe, Australia, and Central and South America, HIAs have become a popular tool to understand the impact of industrial projects on environmental health [83,85,86,87,88]. Due to the lack of resilient infrastructure, safe potable water supplies, climate change, and natural disasters, HIAs are emerging as an important tool for environmental decision-making in low and medium human development index (HDI) countries [83]. The focus on social determinants of health in the US context may not be as useful in low and medium HDI countries, but the focus on equity and environmental justice in the United States in combination with a focus on sustainability could prove beneficial. Furthermore, the rapid HIA because of limited resources and time available may be a good tool in low and medium HDI countries across diverse socio-political contexts [83,85,89]. 

## 5. Conclusions

There were challenges with implementing the rapid HIA including lack of resources, distance between the HIA team and area of concern, and no legal requirement for a health impact assessment. Even with these challenges, the rapid HIA was useful because it provided information to the community about potential negative impacts of the planned processing plant on the environment and human health. The rapid HIA found that the Harim Millsboro processing plant would contribute to air and water pollution, further worsening the health of residents in Millsboro, which has economic and health disparities when compared to the rest of the County and the state of Delaware. Not only would this community be impacted by contaminants released from the new facility that can cause respiratory problems, developmental issues, and cancer, their health risks would also increase because they already suffer from environmental injustice. Residents of Millsboro are overburdened by the presence of other local environmental hazards compared to other populations in the County, which contributes to their cumulative exposure to air and water pollution. 

Furthermore, the use of the HIA emphasized the importance of equity and recognition of the social determinants of health in the decision-making process. The HIA framework provided the team with an opportunity to emphasize how the proposed processing plant would negatively impact the health of vulnerable populations. Additionally, it allowed for more equity by providing a space for residents who would have been adversely impacted by the construction of the facility share their concerns through community engagement that occurred in the scoping and assessment phases of the project. The information was used by the local community in partnership with the Assateague Coastkeeper and Food and Water Watch to launch an educational campaign about the proposed facility and help organize local citizens to be engaged in decision-making about the proposed operation. As a result of the HIA and efforts to educate and organize the residents, Allen Harim Foods terminated its plans to build a poultry processing plant in Millsboro. 

Our study revealed several benefits of a rapid HIA. It can serve as an inexpensive way to increase the environmental health literacy of residents on the negative impacts and possible benefits of industrial activities in their community when time is limited. It can help assess concerns of overburdened and underserved residents when a new land use or operation is proposed. By focusing on social determinants and equity, an HIA can empower populations of concern and help build community capacity for better engagement in local environmental decision-making. It can serve as a guide for citizens as they engage county, state, and federal agencies on environmental health issues and seek assistance from these agencies and non-profit organizations for technical support and solutions. In addition, other communities dealing with similar situations could use the rapid HIA as an approach for assessing their own health concerns associated with industrial animal agriculture, including industrial chicken farming and related activities, such as chicken processing plants when time and financial resources are limited. Through more upfront training for students (and staff) on the HIA framework, additional resources, and making it a mandatory requirement under the law, rapid HIAs can be as impactful as more robust and comprehensive non-rapid HIAs. 

## Figures and Tables

**Table 1:** Summary of Existing Hazards Near Millsboro.

Facility	Distance from Harim Millsboro	Amount of Emissions	Types of Emissions/Pollution	History of Pollution
NRG Indian River Plant	2 miles	279,354 Lbs. (2013) [37]	Chemical—barium, manganese, vanadium, lead [37]	Coal ash dump—groundwater contaminated with arsenic, chromium, and thallium [38] Closed coal-fired units in 2011 and 2013 to meet consent order to limit mercury, sulfur dioxide, and nitrous oxide emissions [39,40,41,42]
Mountaire Millsboro Poultry Processing Plant	2 miles	3167 Lbs [43,44]	Hydrogen sulfide, manganese, copper [33,41]	Received 17 violations from DNREC for exceedances of CO, NH3, nitrous oxides, nitrates, and sulfur oxides [33]
NCR Superfund Site	1 mile	None	Chromium, trichloroethylene (TCE) [41,42]	Wastewater with high levels of chromium treated and stored in unlined pits on site until 1980s; added to NPL in 1987 [41,42,43]
Millsboro TCE Superfund Site	2 miles	None	TCE [44]	2005—remediation occurred because TCE found entering groundwater; bottled water given to residents during this time [45]

**Table 2:** Chemical Information of Pollutants at the Harim Millsboro Processing Plant Site.

Chemical	EPA Standards	Health Effects	EPA Carcinogen Analysis	Concentrations Found at Harim Millsboro
Arsenic *	Maximum Contaminant Level (MCL) of 0.010 mg/L for drinking water	Acute exposure—numbness, nausea, vomiting, or burning sensations in the hands and feet, cardiovascular effects, and fatigue Chronic exposure-dermatological damage	Yes—chronic exposure associated with an increased risk of lung, skin, kidney, bladder, and prostate cancer	Vary from 0.0005–18.2 mg/L [46]
Chloride *	Secondary Maximum Contaminant Level (SMCL) ^2^ of 250 mg/L for drinking water	No known health effects; can cause corrosion in metal pipes, thus increasing amount of heavy metals in water	No	Vary between 12–560 mg/L [46]
Chromium-3 and Chromium-6 *	MCL of 0.1 mg/L for drinking water	Ingestion—Skin irritation Acute inhalation—Respiratory issues Chronic inhalation—Bronchitis, pneumonia, decreased lung function, and nasal septum destruction	Chromium-6 carcinogenic when inhaled and possible carcinogen when ingested	Elevated at site, below MCL in sprayfields [46,47,48]
Cobalt	None	Oral exposure—Nausea, vomiting, vision problems, skin irritation, thyroid damage, heart problems, death Acute inhalation—Decreased lung function, congestion, edema, and hemorrhage Chronic inhalation—Severe respiratory and cardiovascular issues	Not classified for carcinogenicity	Not monitored in public wells; private wells nearby ranged from 0.0018–0.523 mg/L [46]
Hydrogen Sulfide *	None; Occupational Safety and Health Administration (OSHA) has set standards for occupational exposure	Acute exposure—nausea, headache, eye and respiratory tract irritation, death at extremely high concentrations (over 500 ppm) Chronic exposure—impaired vision and sense of smell, dizziness, possible neurological defects	Not classified for carcinogenicity	Not available for site; at nearby Mountaire Processing Plant, 76% of emissions [33]
Nitrate *	MCL of 10 mg/L for drinking water	Methemoglobinemia, neurological issues, death	Not classified nitrate for carcinogenicity	Two wells measured 4.2 and 9.9 mg/L [46]
Particulate Matter (PM_2.5_ and PM_10_) *	PM_2.5_ Primary—12 μg/m^3^ Secondary—15 μg/m^3^ 24 h—35 μg/m^3^ PM_10_ 24 h—150 μg/m^3^	Acute exposure—cardiovascular and respiratory issues Chronic exposure—increased mortality among individuals with chronic heart or lung diseases	Designated carcinogenic for diesel particulate matter; likely to be carcinogenic to humans by inhalation	Not measured at site; nearest monitoring station in Seaford shows above National Ambient Air Quality Standards (NAAQS)
Volatile Organic Compounds (VOCs) *	MCL for Total Trihalomethanes (TTHM) in drinking water is 0.08 mg/L	Acute exposure—nose, throat, and eye irritation; vomiting; nausea; dizziness; headache; worsening of asthma symptoms Chronic exposure to high levels—liver, kidney, and central nervous system damage	Bromoform—Probable human carcinogen Chloroform—likely to be carcinogenic Dibromodichloromethane—not classifiable as carcinogenic	Chloroform, PCE, and TCE most common VOCs, found at site [49]
Trichloroethylene (TCE)	MCL for TCE in drinking water is 0.005 mg/L	Acute exposure via inhalation—dizziness, tiredness, headaches, loss of coordination, cognitive and neurological issues Acute exposure in large quantities in air—unconsciousness or premature death	Likely to be carcinogenic by all routes of exposure	1.2 μg/L [46]

* Common poultry processing plant pollutant.
